# Effects of Simulated Typhoon Stress on Ovarian Function in Wenchang Chickens: An Exploration Based on the Microbiota–Gut–Brain–Ovarian Axis

**DOI:** 10.3390/ani16081241

**Published:** 2026-04-17

**Authors:** Ben Zhang, Lihong Gu, Yangqing Lu, Qicheng Jiang, Xinli Zheng, Tieshan Xu

**Affiliations:** 1 College of Animal Science and Technology, Guangxi University, Nanning 530004, China; 2 Institute of Animal Husbandry and Veterinary Medicine, Hainan Academy of Agricultural Sciences, Haikou 571100, China; 3 Tropical Crops Genetic Resources Institute, Chinese Academy of Tropical Agricultural Sciences, Haikou 571101, China

**Keywords:** typhoon stress, climate extreme, microbiota–gut–brain–ovarian axis, egg production, Wenchang chicken

## Abstract

As a representative form of extreme weather, typhoons inflict widespread and systemic damage, posing a severe threat to the livestock industry. The stress they induce, typhoon stress (TS), is an unavoidable and complex environmental challenge that severely disrupts the ovarian function of Wenchang chickens. In this preliminary study, we employed a two-group comparison design (*n* = 6 per group) integrating behavioral observations, serum biochemical assays, histopathological examinations, and molecular analyses (qPCR, 16S rDNA sequencing, and transcriptome sequencing) to explore the role of the microbiota–gut–brain–ovarian axis (MGBOA) in this process. The findings revealed that TS markedly reduced water intake and locomotor activity, while it elevated serum corticosterone (CORT) and oxidative stress markers. It also induced shifts in gut microbiota composition, including a decrease in *Bacteroides* and an increase in *Escherichia–Shigella*. Furthermore, TS compromises duodenal intestinal barrier integrity, as evidenced by downregulation of the tight junction proteins *TJP1* and *CLDN1*, structural damage to intestinal villi, and a reduced villus-to-crypt ratio. In the hypothalamus, VIP mRNA expression was upregulated, while GHSR expression was downregulated; the expression of the tight junction protein *CLDN5* was also reduced. In the ovary, reproductive potential was suppressed, manifested by a reduction in follicle number and downregulation of *STAR* expression. Ovarian transcriptome analysis highlighted enrichments in pathways associated with inflammation (e.g., Toll-like receptor signaling) and lipid metabolism (e.g., PPAR signaling). These results support the hypothesis that TS impairs egg production via the MGBOA, providing preliminary mechanistic insights into how environmental stressors might disrupt animal productivity through MGBOA-mediated pathways.

## Simple Summary

Typhoons are extreme weather events that are becoming more frequent with climate change. In this study, we investigated how the stress of a typhoon affects the ovarian function of Wenchang chickens, a local breed in Hainan, China. We simulated typhoon conditions in the laboratory and compared chickens exposed to this stress with a control group. We found that the stressed chickens drank less water and were less active. Their bodies showed clear signs of a strong stress response, including changes in their gut bacteria, with a decrease in beneficial bacteria and an increase in harmful ones. This was accompanied by damage to their intestinal lining and signs of inflammation in the brain, which ultimately led to reduced function in their ovaries. Our results suggest that typhoon stress disrupts the important communication pathway between the gut and the brain, which in turn harms reproduction. This research provides a first step toward understanding how extreme weather can threaten livestock productivity, and may help in developing future strategies to protect farm animals from such environmental challenges.

## 1. Introduction

In the context of escalating climate extremes, the frequency and intensity of tropical cyclones (typhoons), particularly in the Northwest Pacific region, have shown a marked increase [1]. As a major typhoon corridor, Hainan Province, China, experiences prolonged annual typhoon seasons, posing significant threats to local ecosystems and agricultural sustainability. On 6 September 2024, Super Typhoon Yagi made landfall in Wenchang City at a historic peak intensity, with maximum winds exceeding 17th scale near its center, resulting in massive economic losses [2]. According to the Wenchang Chicken Industry Association, approximately 13.2 million chickens perished, representing a 60% mortality rate and an economic loss of around 200 million RMB [3]. Beyond immediate physical destruction, typhoons impose a complex environmental stressor (typhoon stress, TS) on livestock, characterized by synergistic effects of violent winds, torrential rain, and acoustic disturbance. This compound stress leads to prolonged declines in the egg production rate of Wenchang chickens, severely suppressing laying hen production performance and highlighting a critical challenge for animal agriculture under climate change.

Stress is a generalized physiological response to external or internal threats, involving integrated neural, endocrine, immune, as well as oxidative and antioxidant, pathways [4]. While mild stress can be buffered by homeostatic mechanisms, severe stress disrupts growth, reproduction, and can lead to multi-organ failure [5]. Notably, the gut–brain axis (GBA) has emerged as a key mediator in stress responses. It constitutes a bidirectional communication network between the gastrointestinal tract and the central nervous system, integrating signals from the gut microbiota, enteric nervous system, and neuroendocrine pathways [6]. Research in laying hens has shown that the gut microbiota plays a vital role in nutrient utilization [7,8], intestinal barrier integrity [9,10], and overall production performance [11,12,13].

Environmental stressors like heat stress can disrupt the GBA, leading to intestinal dysbiosis, inflammation, and altered neuroendocrine signaling, which ultimately impair productivity [14,15]. Although the detrimental effects of conventional stressors like heat on poultry are well-documented, the mechanisms through which complex, multifaceted stressors like typhoons affect livestock via the GBA remain largely unexplored. We hypothesized that TS impairs reproductive function in Wenchang chickens primarily through the disruption of the microbiota–gut–brain–ovarian axis (MGBOA), wherein gut dysbiosis triggers intestinal barrier failure, neuroendocrine alterations, and subsequent ovarian dysfunction. Therefore, this study employed a multi-level approach—combining behavioral, physiological, histopathological, microbiological, and transcriptomic analyses—to investigate potential associations between TS and the ovarian function of Wenchang chickens via the MGBOA. Our findings aim to provide novel insights into the interplay between climate extremes, animal health, and agricultural productivity, contributing to the development of strategies for building resilience in sustainable farming systems.

## 2. Materials and Methods

### 2.1. Experimental Conditions and Animal Handling for Simulated Typhoon Stress

This study utilized 24 healthy 190-day-old Wenchang chickens obtained from Wenchang Longquan Wenchang Chicken Industrial Co., Ltd., Wenchang, Hainan, China. The experiment was conducted in a room measuring 2.87 m × 1.87 m × 3.18 m (length × width × height). The chickens were randomly assigned to either a control group (Group C) or a treatment group (Group T), with 12 chickens per group housed in individual cages. Due to the complex nature of the multi-factor stress simulation and the constraints of the experimental facility, a sample size of six chickens per group was chosen to ensure rigorous individual monitoring while minimizing animal use, in accordance with the 3Rs principle. Following the 12 h simulated typhoon exposure, six chickens from each group were immediately euthanized for physiological and biochemical analysis, while the remaining six chickens from each group were used for subsequent behavioral observation. Group C and Group T were housed under thermoneutral conditions (25 °C, 80% relative humidity) in adjacent rooms of identical dimensions. The experiment was conducted from 19:00 to 07:00, during which no feed, water, or external light sources were provided. To simulate typhoon conditions, Group T was subjected to a 12 h multifactorial stress protocol.

The simulation for Group T concurrently applied (1) aerodynamic stress via industrial fans (Yongji, YJ-012, China) generating a wind speed of ≥12 m/s at chickens’ height; (2) precipitation stress via high-pressure water nozzles (Chezhu Riji, YGQ-079, China) calibrated by five-point positioning within the cage to collect rainwater, yielding a rainfall intensity of 23 ± 2 mm/min; and (3) acoustic stress via a speaker system (Xianke, ST-501, China) playing a pre-recorded typhoon soundtrack with intensities fluctuating between 80 and 120 dB, encompassing a broad frequency spectrum to mimic natural storm dynamics. Following the 12 h exposure, all chickens from both groups were simultaneously sampled for physiological and biochemical analysis. All experimental procedures were approved by the Ethics Committee of the Institute of Animal Science and Veterinary Medicine, Hainan Academy of Agricultural Sciences.

### 2.2. Behavioral Observation and Detection

The cages containing the remaining six chickens from each group (*n* = 6 per group for behavioral observation) were returned to the dry room and immediately replenished with feed and water, and each cage was recorded separately using a mobile phone mounted frontally. Behavioral observations were conducted over a 12 h period from 07:00 to 19:00 on the same day, with 10 min video recording every 2 h, for a total of 6 video recordings per day. The frequency of specific behaviors, including feeding, drinking, locomotion, crouching, and grooming, were quantified by two observers who had been trained on the behavioral definitions in a preliminary test and were blinded to group assignment. The data are expressed as the mean frequency (count) per chicken per minute observation session.

### 2.3. Sample Processing

Blood samples (approximately 3 mL per chicken, collected via wing vein within 3 min of capture) were collected from both groups of Wenchang chickens. Whole-blood samples were stored at room temperature for 2 h and then centrifuged at 3000 rpm/15 min at 2–8 °C. Serum biochemical parameters were quantified using specific commercial assay kits from Servicebio (Wuhan, China) and Nanjing Jiancheng (Nanjing, China), following the respective manufacturers’ instructions. The following serum biochemical indicators were measured: corticosterone (CORT), serotonin (5-HT), aspartate aminotransferase (AST), uric acid (UA), creatine kinase (CK), glycated serum protein (GSP), and total antioxidant capacity (T-AOC). Because CK values exceeded the linear detection range, serum was diluted 1:6 before measurement. Then, cecal contents, duodenum, ovaries, and hypothalamic tissues were collected. The obtained tissue samples were placed in cryopreservation tubes, immediately immersed in liquid nitrogen, and subsequently stored at −80 °C for future experiments.

### 2.4. Paraffin Sectioning and H&E Staining

The tissues were fixed with 4% paraformaldehyde, dehydrated, cleared, paraffin-embedded, and sectioned (5 µm thick). The sections were then dewaxed with xylene and hydrated with a series of graded ethanol solutions and with distilled water. The sections were then stained with hematoxylin and eosin, dehydrated with graded ethanol, cleared with xylene, and mounted using a neutral binder. The prepared sections were scanned using a panoramic slide scanner and imaged using the CaseViewer 2.4 scanning and viewing software.

### 2.5. Real-Time Fluorescent Quantitative PCR Detection

Total RNA was extracted from the hypothalamic, duodenal, and ovarian tissues using an Animal Tissue Total RNA Extraction Kit (TIANGEN, DP451). RNA was then reverse transcribed into cDNA using a Reverse Transcription Kit (TIANGEN, KR118). qRT-PCR was performed using a SuperReal Fluorescent Quantitative Pre-mix Kit (TIANGEN, FP205) on a Bio-Rad CFX96 thermocycler. *GAPDH* was used as the internal control after confirming its stable expression between groups. Expression was quantified using the 2^−ΔΔCt^ method. Target gene primers were synthesized by Beijing Qingke Biotechnology Co., Ltd. (Beijing, China). The primer sequences are detailed in Appendix A.

### 2.6. 16S rDNA Gene Sequencing and Analysis

Total microbial DNA was extracted from cecal contents using a commercial kit (AU46111-96, BioTeke, Beijing, China). DNA concentration was quantified using a Qubit fluorometer (Invitrogen, Carlsbad, CA, USA). The V3–V4 hypervariable region of the total DNA was amplified by PCR using universal primers 341F (5′-CCTACGGGNGGCWGCAG-3′) and 805R (5′-GACTACHVGGGTATCTAATCC-3′). PCR products were purified using AMPure XT beads (Beckman Coulter Genomics, Danvers, MA, USA) and quantified with Qubit (Invitrogen, USA). Purified amplicons were evaluated for quality and concentration using an Agilent 2100 Bioanalyzer (Agilent, Santa Clara, CA, USA) and the Illumina library quantification kit (Kapa Biosciences, Woburn, MA, USA). Libraries were pooled in equimolar amounts, denatured into single strands, and sequenced on an Illumina NovaSeq 6000 platform (PE250) using the NovaSeq 6000 SP Reagent Kit (500 cycles) by LC-Bio Technology Co., Ltd. (Hangzhou, China).

Raw sequencing data were demultiplexed based on barcodes, and primer and barcode sequences were removed using cutadapt (v1.9). Paired-end reads were merged using FLASH (v1.2.8) with parameters “-m 10 -M 100 -x 0.25 -t 1 -z”. Quality filtering was performed with fqtrim (v0.94) using a sliding-window algorithm (window size 100 bp, average quality threshold <20); reads shorter than 100 bp or containing >5% ambiguous bases (“N”) were discarded. Chimeric sequences were identified and removed using Vsearch (v2.3.4) with default parameters. Denoising and generation of amplicon sequence variants (ASVs) were carried out with the DADA2 plugin in QIIME 2, and singleton ASVs (those with a total abundance of 1 across all samples) were removed. Taxonomic assignment of ASVs was performed against the SILVA database (Release 138) with a confidence threshold of 0.7, and against the NT-16S database (Release 20230718) using the criteria of minimum identity ≥ 90%, minimum coverage ≥ 80%, and maximum e-value ≤ 1 × 10^−5^.

Alpha diversity indices (observed species, Shannon, Simpson, Chao1, goods_coverage, Pielou’s evenness, and ACE) were calculated using QIIME2. Beta diversity was assessed based on weighted/unweighted UniFrac, Jaccard, and Bray–Curtis distances. Differential abundance of bacterial genera between groups was evaluated using the Wilcoxon rank-sum test (for two groups) or Kruskal–Wallis test (for multiple groups), with significance set at *p* < 0.05. Linear discriminant analysis effect size (LEfSe) was applied to identify biomarkers with LDA score ≥ 3.0 and *p* < 0.05. All statistical analyses and visualizations were performed in R (v3.4.4).

### 2.7. Reference Transcriptome Sequencing and Analysis

Total RNA was extracted from samples using Trizol reagent (Thermo Fisher, 15596018), followed by the enrichment of mRNA with oligo (dT) beads and fragmentation. The sequencing library was constructed through cDNA synthesis, end repair, adapter ligation, and PCR amplification, and was subsequently sequenced on an Illumina NovaSeq 6000 platform to generate paired-end reads. The raw data were then processed by removing adapters and low-quality bases to obtain clean reads, which were aligned to the reference genome using HISAT2. Gene expression levels were quantified and assembled using StringTie, and gene differential expression analysis was performed by DESeq2 software (vertion 1.50.2) between two different groups (and by edgeR between two samples). Genes with a false discovery rate (FDR) below 0.05 and absolute fold change ≥ 2 were considered differentially expressed genes. Finally, functional interpretation of the results was achieved through Gene Ontology (GO) and Kyoto Encyclopedia of Genes and Genomes (KEGG) pathway enrichment analyses.

### 2.8. Statistical Analysis

All statistical analyses were performed using GraphPad Prism 8.0 software. Results are presented as mean ± standard error. Data normality and homogeneity of variances were tested using the Shapiro–Wilk test and the F-test, respectively. If the assumptions of normality and homogeneity of variance were met, intergroup differences were analyzed using Student’s *t*-test. Otherwise, the non-parametric Mann–Whitney U test was applied. Results were considered statistically significant at *p* < 0.05.

## 3. Results

### 3.1. Effects of Typhoon Stress on the Behavioral Characteristics of Wenchang Chickens

**Figure 1:**
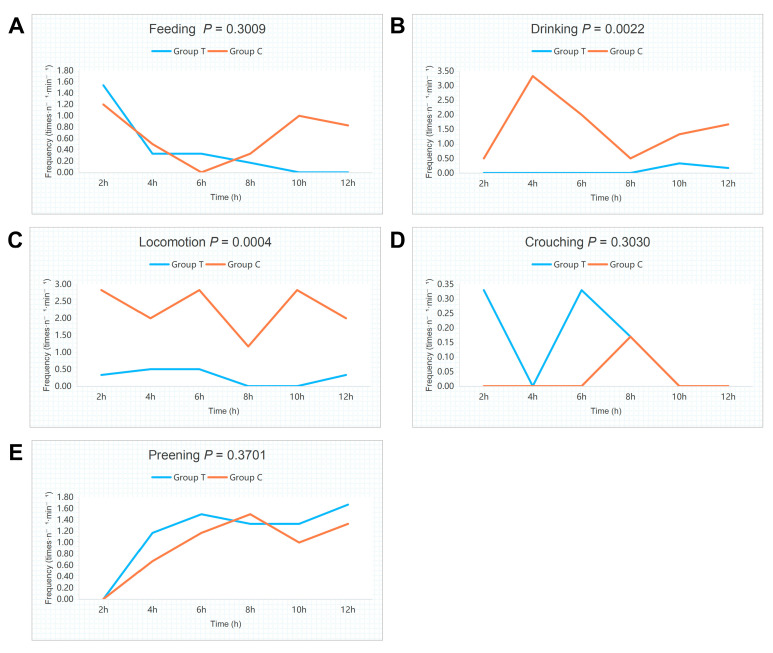
Effects of TS on the frequency of various behaviors in Wenchang chickens. (**A**) Feeding behavior. (**B**) Drinking behavior. (**C**) Locomotor behavior. (**D**) Crouching behavior. (**E**) Preening behavior.

As shown in Figure 1, TS significantly affected the behavioral characteristics of Wenchang chickens. Compared with Group C, Group T showed significantly lower total drinking and locomotor frequencies throughout the observation period (*p* < 0.01 and *p* < 0.001). Group T exhibited a decreasing trend in feeding frequency, whereas crouching and preening frequency showed increasing trends. However, neither difference reached statistical significance (*p* > 0.05). During the initial stress phase, drinking was completely suppressed in Group T, and the locomotor frequency gradually declined. Group C maintained normal drinking and physiological activities. At 8 h post-stress, drinking behavior was observed in Group T, although its frequency remained lower than that in Group C at the same time point. Meanwhile, locomotor frequency decreased to nearly zero and did not recover until after 10 h. These results indicated that although Group T exhibited adaptive behavioral processes, behavioral suppression persisted following stress exposure. Please see Table S1 for detailed behavioral records of each time period.

### 3.2. Effects of Typhoon Stress on Hepatic, Renal, and Cardiac Function Indicators and Oxidative Stress

Changes in CORT concentrations are key parameters for evaluating stress. As shown in Table 1, compared to Group C, the serum CORT concentrations in Wenchang chickens in Group T were significantly higher (*p* < 0.01). To evaluate the effect of TS on serum biochemistry of Wenchang chickens, we tested the collected serum. AST and CK concentrations in Group T increased significantly (*p* < 0.01), rising by 72.9% and 272.0%, respectively, compared with those in Group C. UA concentrations were significantly elevated (*p* < 0.05) by 156.9%. GSP concentrations decreased significantly (*p* < 0.05) by 33.6%. Additionally, serum T-AOC in Group T was also significantly increased (*p* < 0.05). These results suggested that TS may trigger an intense neuroendocrine response, which could potentially lead to indicators of multi-organ dysfunction in the liver, myocardium, and kidneys. This dysfunction was accompanied by alterations in glucose metabolism and increased oxidative stress.

### 3.3. Typhoon Stress Causes Damage to Duodenum, Hypothalamus, and Ovarian Tissues in Wenchang Chickens

**Figure 2:**
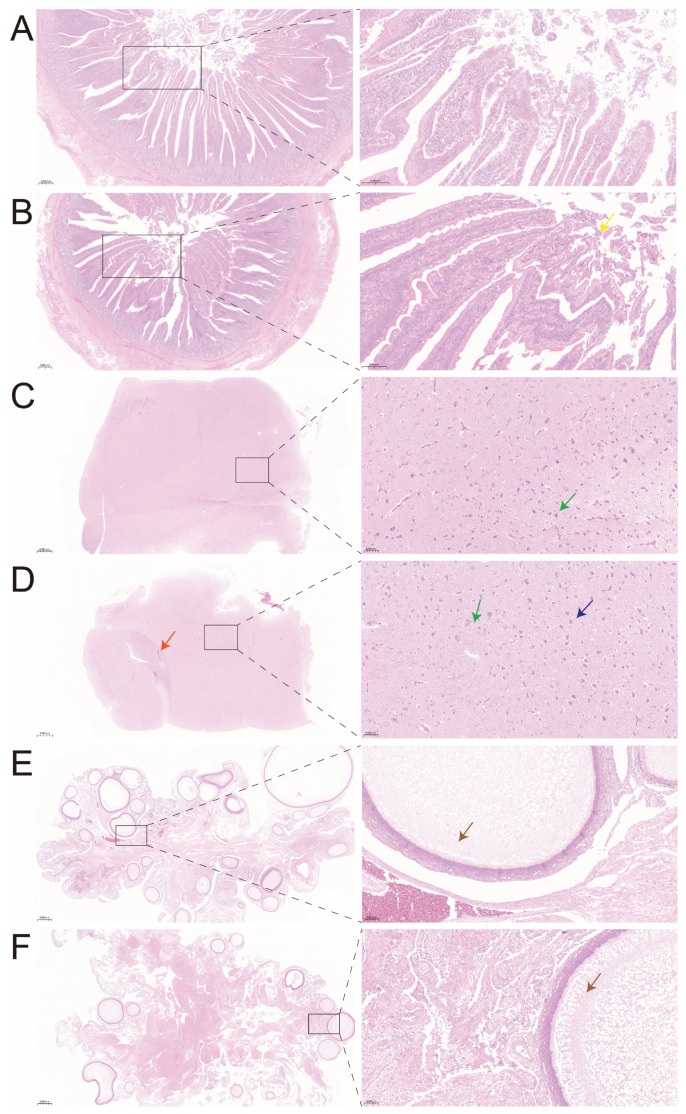
Effects of typhoon stress on morphology of the duodenum, hypothalamus, and ovary. (**A**) H&E staining of the duodenum in Group C. (**B**) H&E staining of the duodenum in Group T. (**C**) H&E staining of the hypothalamus in Group C. (**D**) H&E staining of the hypothalamus in Group T. (**E**) H&E staining of the ovary in Group C. (**F**) H&E staining of the ovary in Group T.

Through H&E staining of the duodenum, hypothalamus, and ovary of Wenchang chickens, it was observed that compared with Group C, Group T exhibited shedding of villous epithelial cells in the duodenum after TS (Figure 2B, yellow arrow), edema of myocytes in the muscular layer, pale cytoplasm and loosened architecture, indicating epithelial barrier damage and cellular stress. Interstitial microvacuolation was observed in the hypothalamus of both groups (Figure 2C,D, green arrows). In Group T, hypothalamic neurons appeared shrunken (Figure 2D, blue arrow) with blurred nucleocytoplasmic boundaries, accompanied by vascular congestion (Figure 2D, orange arrow), suggestive of neuronal apoptosis and microcirculatory disturbance. Cystic dilation of follicles was present in the ovaries of both groups (Figure 2E,F, brown arrows). However, Group T showed a reduced number of follicles and a scarcity of primary follicles, consistent with impaired development of germ cells following TS.

**Figure 3:**
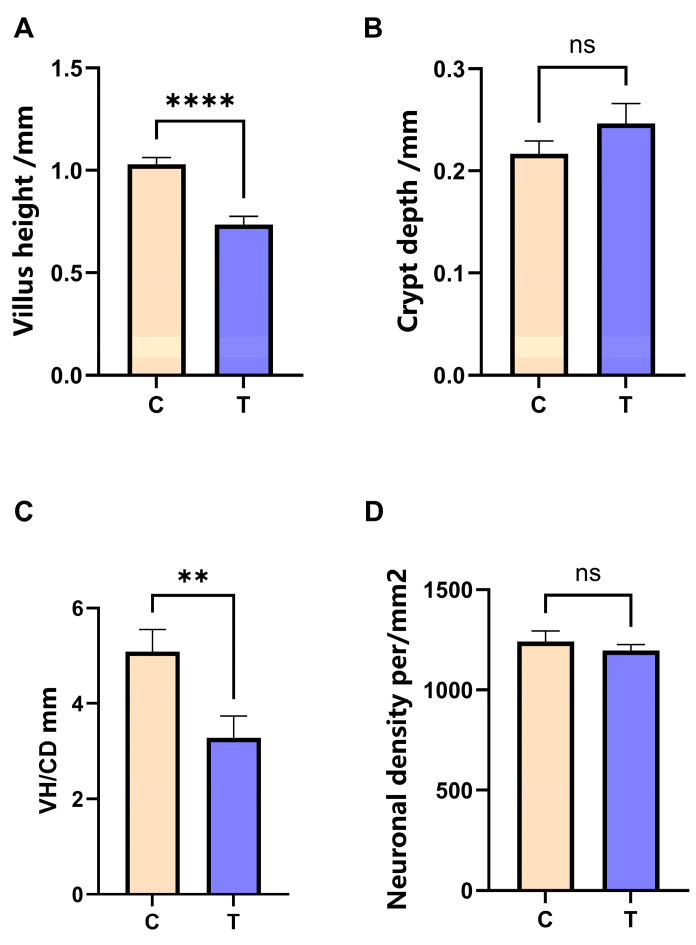
Typhoon stress-induced damage to duodenal, hypothalamic, and ovarian tissues in Wenchang chickens. (**A**) Duodenal villus height. (**B**) Duodenal crypt depth. (**C**) Duodenal villus-to-crypt ratio. (**D**) Number of neurons per unit field of view in the diencephalon. ** *p* < 0.01, **** *p* < 0.0001. ns, not significant.

Histomorphometric analysis revealed that villus height was significantly lower in Group T than in Group C (*p* < 0.01; Figure 3A). Concurrently, crypt depth exhibited an increasing trend in Group T (Figure 3B). The combination of reduced villus height and deepened crypts resulted in a significant decrease in the villus-to-crypt ratio (*p* < 0.05; Figure 3C). Furthermore, a decrease in the number of neurons per unit field was observed in Group T (*p* > 0.05; Figure 3D), indicative of morphological changes associated with neuronal damage.

### 3.4. Typhoon Stress Induces Changes in the Gut Microbiota of Wenchang Chickens

**Figure 4:**
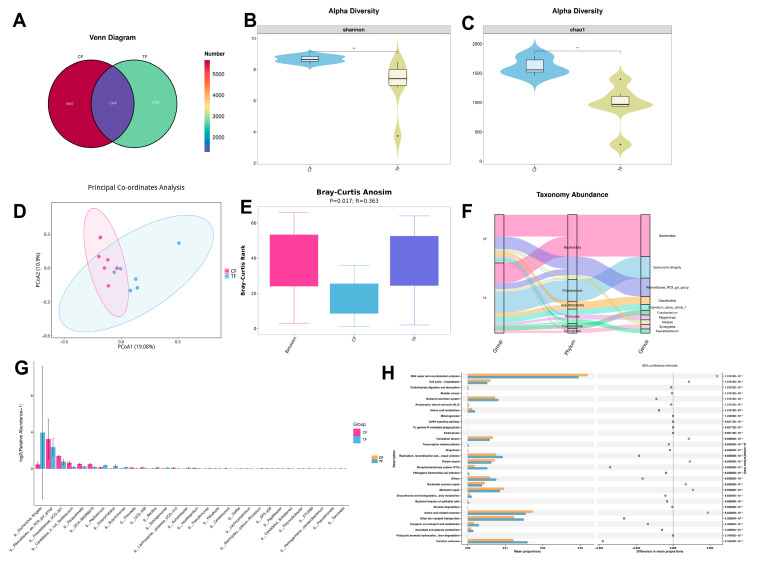
The effects of typhoon stress on the gut microbiota of Wenchang chickens. CF represents the cecal contents of Group C, and TF represents the cecal contents of Group T. (**A**) Venn diagram of ASVs. (**B**) Shannon index. (**C**) Chao1 index. (**D**) PCoA plot (Bray–Curtis). (**E**) ANOSIM similarity analysis. (**F**) Sankey plot of top 10 abundant taxa (phylum and genus). (**G**) Genus-level composition bar plot. (**H**) PICRUSt2 functional prediction (KEGG Level 3). ** *p* < 0.01.

Changes in the intestinal microbiota of Wenchang chickens after TS were analyzed using 16S rDNA gene sequencing. The total number of shared amplicon sequence variants (ASVs) in the intestinal microbiota of Groups C and T after TS was 1304. There were 5656 ASVs unique to Group C and 2700 ASVs unique to Group T (Figure 4A). Alpha diversity analysis revealed a decrease in gut community richness and diversity in Wenchang chickens after exposure to TS (Figure 4B,C). Beta diversity analysis revealed differences in the species-level gut microbiota composition between the two groups (Figure 4D,E). Compared to Group C, the abundance of *Bacteroides* in Group T, although remaining relatively high in both groups, exhibited a decreasing trend, whereas *Escherichia-Shigella* showed a significant increase (*p* < 0.01) (Figure 4F,G). Functional prediction using PICRUSt2 revealed significant differences in the abundance of metabolic functions between Groups C and T (Figure 4H). Group T exhibited lower proportions of core pathways, such as carbohydrate digestion, amino acid metabolism, and inorganic ion transport, than Group C. This may suggest relatively weaker basal energy metabolism. Meanwhile, Group T exhibited higher proportions of pathways, such as melanin synthesis, secondary metabolite synthesis/degradation, and detoxification of exogenous substances. These results suggest that TS may induce alterations in the composition and structure of the gut microbiota, which could influence metabolic processes.

### 3.5. Effects of Typhoon Stress on Functional Gene Expression in Duodenum, Hypothalamus, and Ovary Tissues of Wenchang Chickens

**Figure 5:**
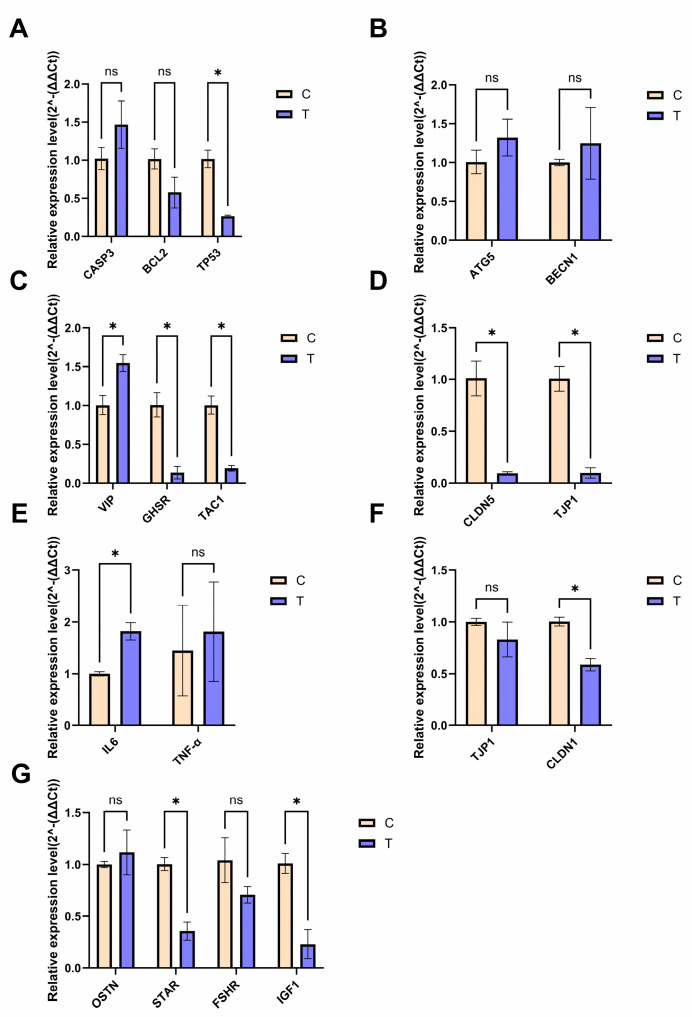
Effects of typhoon stress on functional gene expression in different tissues of Wenchang chickens. (**A**) Apoptosis-related genes in hypothalamus. (**B**) Autophagy-related genes in hypothalamus. (**C**) Neurotransmitter-related genes in hypothalamus. (**D**) Tight junction-related genes in hypothalamus. (**E**) Inflammation-related genes in duodenum. (**F**) Tight junction-related genes in duodenum. (**G**) Egg-laying performance-related genes in ovary. *p* < 0.05 (*), ns, not significant.

This experiment examined the expression levels of relevant functional genes in the duodenum, hypothalamus, and ovarian tissues of Wenchang chickens (Figure 5). Analysis of apoptosis marker genes caspase 3 (*CASP3*), tumor protein p53 (*TP53*), and *BCL2* apoptosis regulator (*BCL2*) revealed that, in Group T, *TP53* was significantly downregulated (*p* < 0.01) in the hypothalamus; *BCL2* showed a downward trend (*p* > 0.05); and *CASP3* exhibited an upward trend (*p* > 0.05). Autophagy-related 5 (*ATG5*) and Beclin 1 (*BECN1*) showed increased expression, although the difference was not statistically significant (*p* > 0.05). Vasoactive intestinal peptide (*VIP*) was significantly upregulated (*p* < 0.01), whereas growth hormone secretagogue receptor (*GHSR*) and tachykinin precursor 1 (*TAC1*) were significantly downregulated (*p* < 0.01). Claudin 5 (*CLDN5*) and tight junction protein 1 (*TJP1*) were also significantly downregulated (*p* < 0.01). Overall, these results indicated an imbalance in pro-apoptotic/anti-apoptotic signaling, neuroendocrine dysregulation, and blood–brain barrier disruption.

In Group T, interleukin 6 (*IL6*), a pro-inflammatory factor, was significantly upregulated in the duodenum (*p* < 0.05), whereas lipopolysaccharide-induced TNF factor (*TNF-α*) showed an increasing trend (*p* > 0.05). In addition, the expression of claudin 1 (*CLDN1*) was significantly downregulated (*p* < 0.01). These results suggested that TS exacerbated duodenal inflammatory responses and formed a co-damage pattern of the gut–brain barrier, alongside hypothalamic *TJP1* downregulation.

In the ovaries in Group T, the acute regulatory protein gene for steroid synthesis (*STAR*) and insulin-like growth factor gene (*IGF1*) were significantly downregulated (*p* < 0.05). This suggested that TS may directly impair egg-laying potential by inhibiting steroid synthesis and growth signaling. The downward trend in follicle-stimulating hormone receptor (*FSHR*) expression (*p* > 0.05) and the upward trend in osteogenic (*OSTN*) expression suggested multifactorial regulation of the reproductive axis.

### 3.6. Transcriptome Expression Analysis of the Hypothalamus in Typhoon Stress

**Figure 6:**
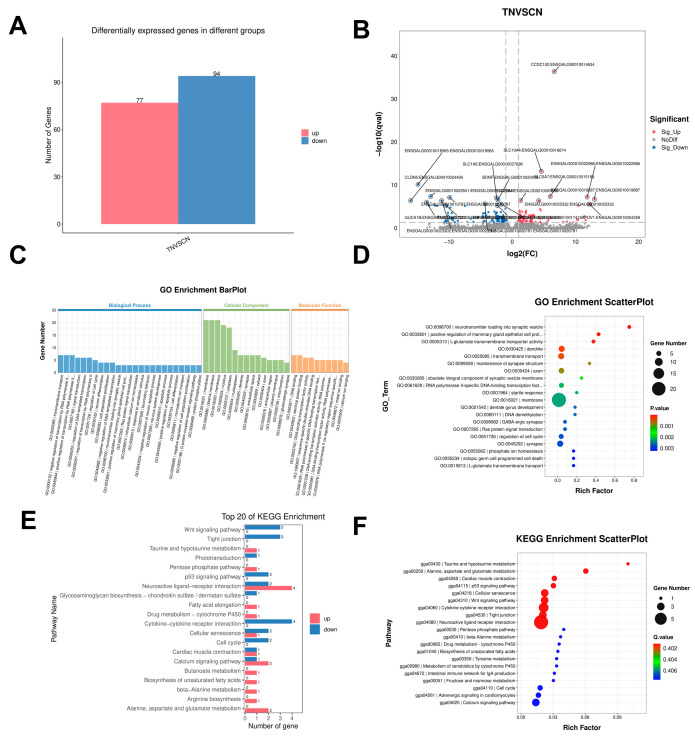
Transcriptomic profiling of the hypothalamus in Wenchang chickens. TN, hypothalamic tissue from Group T; CN, hypothalamic tissue from Group C. (**A**) Number of differentially expressed genes (DEGs) between groups. (**B**) Volcano plot of top 20 DEGs. (**C**) GO enrichment classification (BP: top 25; CC: top 15; MF: top 10). (**D**) Bubble plot of top 20 GO terms (Q-value). (**E**) Top 20 KEGG pathways with DEG distribution. (**F**) Bubble plot of top 20 KEGG pathways (Q-value).

Analysis of the hypothalamic tissue transcriptome identified 171 differentially expressed genes, including 77 upregulated and 94 downregulated genes (Figure 6A,B). These differentially expressed genes showed significant enrichment in GO terms, such as “neurotransmitter loading into synaptic vesicles” (GO:0098700), “positive regulation of mammary epithelial cell proliferation” (GO:0033601), and “glutamate transmembrane transporter activity” (GO:0005313) (Figure 6C,D). KEGG enrichment analysis revealed significant enrichment in pathways such as neuroactive ligand–receptor interaction (gga04080); metabolism of alanine, aspartic acid, and glutamic acid (gga00250); and cytokine–cytokine receptor interaction (gga04060) (Figure 6E,F). The key genes enriched in the hypothalamus and identified in these pathways were *MC4R* (gga04080), *ASS1* (gga00250), and *IL1RL1* (gga04060).

### 3.7. Ovarian Transcriptome Analysis

**Figure 7:**
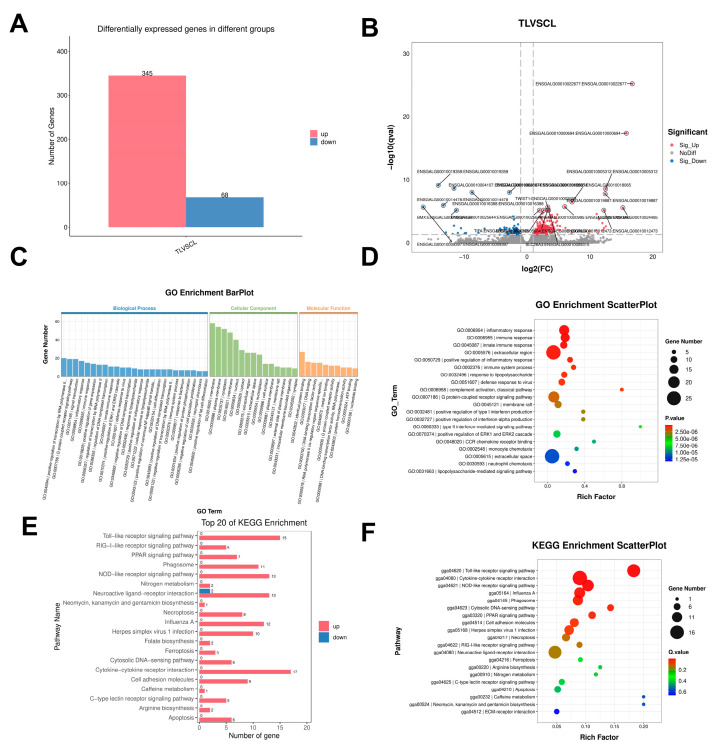
Transcriptomic profiling of the ovary in Wenchang chickens. TL, ovarian tissue from Group T; CL, ovarian tissue from Group C. (**A**) Number of differentially expressed genes (DEGs) between groups. (**B**) Volcano plot of top 20 DEGs. (**C**) GO enrichment classification (BP: top 25; CC: top 15; MF: top 10). (**D**) Bubble plot of top 20 GO terms (Q-value). (**E**) Top 20 KEGG pathways with DEG distribution. (**F**) Bubble plot of top 20 KEGG pathways (Q-value).

Analysis of the ovarian tissue transcriptome identified 413 differentially expressed genes in TS-treated chicken, including 345 upregulated and 68 downregulated genes (Figure 7A,B). These differentially expressed genes showed significant enrichment in GO terms, including inflammatory process (GO:0006954), immune response (GO:0006955), innate immunity (GO:0045087), and extracellular region (GO:0005576) (Figure 7C,D). Ovarian KEGG pathways showed significant enrichment in the Toll-like receptor signaling pathway (gga04620), cytokine–cytokine receptor interaction pathway (gga04060), and NOD-like receptor signaling pathway (gga04621) (Figure 7E,F). Key genes were identified among the enriched pathways, including *TLR4*, *FABP4*, *IRF7*, *CASP18*, *GRP*, and *WIF1*.

### 3.8. qPCR Validation of Key Differentially Expressed Genes

**Figure 8:**
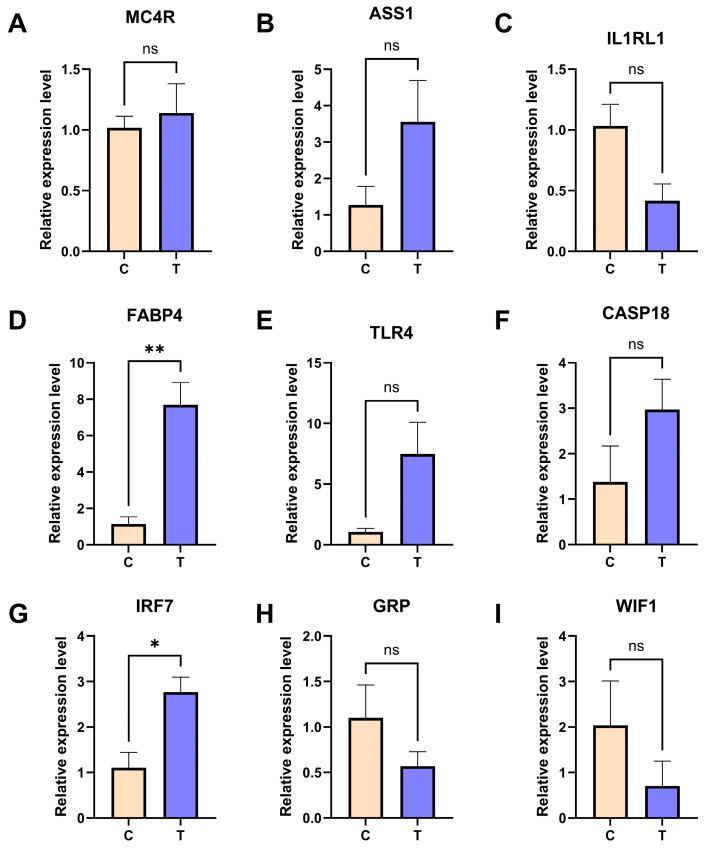
Validation of differentially expressed genes (DEGs) in the ovary and hypothalamus of Wenchang chickens by mRNA relative expression. (**A**–**C**) DEGs in ovarian tissue; (**D**–**I**) DEGs in hypothalamic tissue. *p* < 0.05 (*), *p* < 0.01 (**), ns, not significant.

Eleven key target genes that were significantly enriched (involving neuroactive ligand–receptor interactions; alanine, aspartate, and glutamate metabolism; cytokine–cytokine receptor interactions; Toll-like receptor signaling pathways; and peroxisome proliferator-activated receptor [PPAR] signaling pathways) were selected from the results of the enrichment analysis for qPCR validation. In ovarian tissues, the expression of melanocortin 4 receptor (*MC4R*), argininosuccinate synthase 1 (*ASS1*) and interleukin 1 receptor-like 1 (*IL1RL1*) was validated. The results showed that, compared with the control group, the mRNA expression levels of MC4R and ASS1 in the ovarian tissues of Group T exhibited an upward trend, whereas IL1RL1 showed a downward trend. These expression patterns were fully consistent with the transcriptome sequencing data (FPKM) (Figure 8A–C), although the differences for these three genes did not reach statistical significance (*p* > 0.05). In hypothalamic tissues, the results showed that the expression of fatty acid-binding protein 4 (*FABP4*), Toll-like receptor 4 (*TLR4*), caspase 18 (*CASP18*), and interferon-regulatory factor 7 (*IRF7*) exhibited upward trends, whereas gastrin-releasing peptide (*GRP*) and WNT-inhibitory factor 1 (*WIF1*) exhibited downward trends, all of which were consistent with the sequencing trends (Figure 8D–I). Among these, the expression levels of *FABP4* (*p* < 0.001) and *IRF7* (*p* < 0.01) were significantly higher in Group T than Group C. Although the changes in the remaining genes did not reach significant levels (*p* > 0.05), their expression trends remained consistent with the sequencing results.

## 4. Discussion

Typhoons are frequent and complex extreme climate stressors in coastal regions. They are a key environmental factor threatening intensive farming operations and usually lead to declines in poultry production through the combined effects of strong winds, heavy rainfall, and intense acoustic disturbances. Company records from Wenchang Longquan Wenchang Chicken Industrial Co., Ltd. showed that following Typhoon Yagi, the egg production rate of Wenchang chickens dropped by approximately 70%, and it took more than 20 days for their laying performance to return to normal levels. However, the mechanisms by which complex stressors, such as typhoons, affect chicken egg production are unclear. Our preliminary findings reveal that TS may disrupt ovarian function through a cascade potentially involving gut microbiota dysbiosis, intestinal barrier damage, neuroinflammation, and local ovarian inflammation, offering a mechanistic basis for the observed long-term production losses. From a broader environmental perspective, this MGBOA-mediated pathway offers a plausible mechanism by which complex climate extremes might disrupt animal physiology and agricultural productivity.

TS appears to impact the behavioral performance of Wenchang chickens. Under heat stress, poultry exhibit behavioral changes, including panting, increased water intake, reduced feed consumption, drooping of wings, decreased activity, and frequent crouching [16]. Our data show that typhoon-stressed Wenchang chickens exhibited significantly suppressed drinking and locomotor frequencies. This behavioral profile, accompanied by increased crouching and preening, suggests that typhoon stress triggers a distinct response likely rooted in psychological tension rather than thermoregulation. This state of tension is likely to affect neural signaling within brain tissue.

Physiological testing of typhoon-stressed Wenchang chickens revealed significantly elevated serum CORT levels, indicating direct activation of the HPA axis in response to TS. As a key neurotransmitter in the GBA, 5-HT is synthesized by intestinal chromaffin cells at a rate of 95% and regulates stress responses bidirectionally via the GBA [17]. In this study, although 5-HT levels showed an increasing but non-significant trend, this pattern aligns with reports that acute stress may promote 5-HT secretion to buffer excessive sympathetic activation [18], contrasting with chronic stress models that often show reduced 5-HT [19]. Heat stress induces the release of oxidative stress markers, such as oxygen free radicals and damaged enzymes, such as AST, in poultry, thereby affecting glucose and lipid metabolism [20]. The results of this study demonstrated a significant elevation in serum AST, CK, and UA concentrations. These findings are consistent with the results reported by Tang et al. [21] on heat stress-induced liver damage in broiler chickens and by Wang et al. on the effects of oxidative stress on serum biochemical indicators in broiler chickens [21,22]. These results suggest that TS may cause multi-organ damage. Collectively, these changes indicate excessive activation of the HPA axis. It is plausible that this physiological shift redirects energy toward survival maintenance, which may consequently suppress reproductive function, as evidenced by the observed ovarian molecular and morphological changes [23].

The gut–brain axis is a bidirectional regulatory system in which the gastrointestinal tract and central nervous system interact via complex networks. Conventional stress accelerates intestinal repair by reducing villus height and increasing the crypt depth [24]. Histological analysis revealed that TS significantly compromised intestinal mucosa structure in Wenchang chickens, as evidenced by a marked reduction in the villus height to crypt depth ratio (*p* < 0.05). This structural alteration indicates an impairment of the intestine’s absorptive function. QPCR analysis revealed extremely significant upregulation of duodenal *IL-6* expression and downregulation of tight junction genes *TJP1* and *CLDN1* (*p* < 0.01), indicating exacerbated intestinal inflammation and compromised barrier function [25]. The reduction in crypt depth may result from inflammation, which directly inhibits stem cell proliferation [26,27]. Concurrently, vasoactive intestinal peptide (*VIP*) was significantly upregulated (*p* < 0.05), and growth hormone secretagogue receptor (*GHSR*) was markedly downregulated (*p* < 0.01) in the hypothalamic tissue, jointly driving the sustained hyperactivity of the HPA axis. Meanwhile, the significant downregulation (*p* < 0.01) of the blood–brain barrier key genes *CLDN5* and *TJP1* further exacerbates the neuroinflammatory microenvironment, potentially inhibiting the reproductive axis function [28]. Interestingly, within this neuroinflammatory context, we observed a significant downregulation of *TP53* (*p* < 0.01) in the hypothalamus. While stress is typically associated with p53-mediated apoptosis, this finding may reflect tissue-specific regulatory dynamics. In the hypothalamus, the degree of stress may have reached an adaptive threshold that prioritizes survival and functional maintenance over the initiation of apoptotic programs. It should also be noted that these samples were collected under controlled laboratory simulations of typhoon conditions, which may not fully replicate the intensity and complexity of a natural typhoon event; further studies under field conditions are warranted to validate these observations. The significant downregulation (*p* < 0.05) of the key gene *STAR*, which is involved in steroid synthesis in ovarian tissue [29], along with the inhibitory trend of the follicular development-related gene *FSHR*, supports this possibility [30,31].

An increasing number of reports indicate that gut microbiota homeostasis can directly influence immune function, metabolism, and brain physiology in animals [32]. *Bacteroides* and *Escherichia-Shigella* species affect neural activity by synthesizing 5-HT, gamma-aminobutyric acid, and short-chain fatty acids [33]. Zhang et al. discovered that *Bacteroides* species can alleviate diarrhea symptoms in rats by modulating the gut microbiota [34]. In humans, *Bacteroides* protect the gut from pathogens, provide nutrients, and offer cancer prevention benefits [35]. Research on *Escherichia coli* and *Shigella* species suggests that these bacteria may contribute to kidney disease and anxiety disorders [36,37]. Research has indicated an interaction network, the “gut–testicular axis”, between the gut microbiota and the male reproductive system, which reveals its impact on male reproductive function [38,39]. Our findings revealed significant disruption of the gut microbiota, including reduced α-diversity, decreased abundance of potentially probiotic *Bacteroides*, and increased proportion of proinflammatory *Escherichia-Shigella* species. This gut dysbiosis appears to be the initiating factor that compromises intestinal barrier integrity, as the increased abundance of gram-negative *Escherichia-Shigella* produces lipopolysaccharide (LPS) that can activate *TLR4* signaling on intestinal epithelial cells, thereby triggering a local inflammatory cascade that directly links the observed microbial shift to the significant upregulation of duodenal *IL-6* (*p* < 0.05) detected in our qPCR analysis. In ovarian tissue, the key steroid synthesis gene *STAR* was significantly downregulated (*p* < 0.05), whereas the follicle development-related gene *FSHR* showed an inhibitory trend. Ovarian transcriptome enrichment analysis revealed the activation of inflammatory pathways, such as those involving Toll-like and NOD-like receptors. These alterations suggest a local inflammatory milieu that could potentially impair the environment necessary for follicular development and steroidogenesis [40].

Subsequent qPCR validation confirmed the transcriptome trends, including the upregulation of hypothalamic *MC4R* and *ASS1* and downregulation of *IL1RL1*, as well as opposing expression changes in ovarian genes such as *TLR4*, *FABP4*, *IRF7*, *CASP18*, *GRP*, and *WIF1*. These validated changes offer molecular insights into the observed phenotypes. In the hypothalamus, the pronounced upregulation of *MC4R*, a key receptor for appetite suppression, provides a direct explanation for the reduced feeding behavior and consequent energy deficiency in stressed chickens [41]. Concurrently, the upregulation of *ASS1*, a pivotal enzyme in arginine metabolism, suggests a stress-induced reprogramming of energy substrates, potentially impacting nitric oxide (NO) synthesis [42,43]. This may contribute to neuroinflammatory processes, which could be exacerbated by the downregulation of the anti-inflammatory receptor *IL1RL1*, indicating a compromised tissue repair capacity in the hypothalamic region [44,45]. In the ovaries, a coordinated pattern emerged indicative of innate immune activation and tissue dysfunction. The significant upregulation of *TLR4* and *IRF7* points to a stress-triggered local inflammatory response [46,47]. The inflammatory milieu was further underscored by the upregulation of *CASP18*, which suggests pyroptosis activation [48]. The dysregulation of lipid metabolism-related *FABP4*, consistent with the downregulation of the steroid synthesis gene *STAR*, may disrupt the follicular environment [49]. This pathogenic cascade is compounded by the downregulation of *WIF1*, an inhibitor of the WNT pathway, potentially altering critical signaling for normal cell proliferation and differentiation [50].

Several limitations, however, must be acknowledged. First, the relatively small sample size (*n* = 6 per group) may limit the statistical power and generalizability of the findings, potentially overlooking subtle but biologically meaningful differences. Additionally, the cross-sectional design with a single sampling time point captures only the acute phase of typhoon stress, leaving the temporal dynamics of recovery and chronic adaptation unexplored. Consequently, while our findings reveal immediate disruptions in ovarian function, their direct translation to long-term egg-laying performance remains inferential. Beyond these design constraints, the laboratory-based simulation—although multifactorial—cannot fully replicate the complex, unpredictable nature of real-world typhoon events. Further research should therefore incorporate larger cohorts, longitudinal sampling to trace the trajectory of stress responses, and field-based validations under authentic typhoon conditions. Such efforts will be essential to confirm the ecological relevance of the MGBOA and to strengthen the translational value of these preliminary insights for climate-resilient livestock management.

In summary, our multi-omics findings delineate a cascade of associated changes whereby TS is linked to gut microbiota dysbiosis, compromised intestinal and blood–brain barrier integrity, induced neuroinflammation, and ultimately suppressed ovarian function in Wenchang chickens. While this pattern differs from some pathways activated by conventional heat stress, further research is needed to confirm its uniqueness. The implications of this study extend beyond a single typhoon event or poultry species. It provides a conceptual framework for understanding how intense environmental stressors associated with climate change can disrupt agricultural productivity by interfering with fundamental neuroendocrine-immune pathways via the MGBOA. As climate variability increases, developing resilience in livestock production systems becomes paramount for global food security. This research highlights the MGBOA as a critical target for developing intervention strategies, such as probiotic supplementation or dietary modulation, to enhance stress resilience in farm animals. By elucidating potential molecular and physiological links between a changing climate and animal health, this work contributes to the broader goal of building sustainable and adaptive agricultural ecosystems in the face of global environmental change.

## 5. Conclusions

Simulated typhoon stress impairs ovarian function in Wenchang chickens through disruption of the microbiota–gut–brain–ovarian axis. This stressor induces behavioral suppression, elevates serum corticosterone and oxidative stress markers, and causes gut microbiota dysbiosis characterized by decreased *Bacteroides* and increased *Escherichia–Shigella*. Intestinal barrier damage and neuroinflammation subsequently develop. Ovarian dysfunction is evidenced by reduced follicle numbers and downregulated *STAR* expression. Transcriptomic analysis reveals activation of inflammatory pathways such as Toll-like receptor signaling and lipid metabolism pathways including PPAR signaling. These findings provide mechanistic insights into climate-driven reproductive losses in poultry.

## Figures and Tables

**Table 1:** Detection of serum biochemical markers in organs from typhoon stress-induced chickens (*n* = 6 per group). Different lowercase letters indicate significant differences (*p* < 0.05).

Items	Groups		*p*-Value
	C	T	
Corticosterone (CORT), ng/mL	4.24 ± 0.40 ^b^	31.57 ± 1.25 ^a^	0.0022
Serotonin (5-HT), ng/mL	29.06 ± 3.73	30.79 ± 3.77	0.7544
Alanine aminotransferase (ALT), U/L	0.89 ± 0.30	2.21 ± 0.38	0.0522
Aspartate aminotransferase (AST), U/L	323.60 ± 22.88 ^b^	559.50 ± 21.75 ^a^	0.0017
Urea (UREA), mmol/L	0.51 ± 0.12	1.04 ± 0.24	0.2000
Uric acid (UA), μmol/L	167.00 ± 15.99 ^b^	429.10 ± 80.14 ^a^	0.0326
Creatine kinase (CK), U/L	500.50 ± 122.2 ^b^	1862.00 ± 174.6 ^a^	0.0031
Lactate dehydrogenase (LDH), U/L	737.70 ± 96.61	1067.00 ± 138.4	0.1224
Glucose (GLU), mmol/L	10.86 ± 0.48	9.85 ± 0.19	0.1198
Glycated serum protein (GSP), mmol/L	1.16 ± 0.072 ^a^	0.77 ± 0.05 ^b^	0.0125
Malondialdehyde (MDA), μmol/L	8.10 ± 0.99	6.92 ± 0.99	0.4455
Total antioxidant capacity (T-AOC), mM	0.47 ± 0.01 ^b^	0.73 ± 0.08 ^a^	0.0424

## Data Availability

The raw sequence data reported in this paper have been deposited in the Supplementary Materials, and in the Genome Sequence Archive (Genomics, Proteomics & Bioinformatics 2025) in National Genomics Data Center (Nucleic Acids Res 2025), China National Center for Bioinformation/Beijing Institute of Genomics, Chinese Academy of Sciences (GSA: CRA030941, GSA: CRA030924, GSA: CRA031660) that are publicly accessible at https://ngdc.cncb.ac.cn/gsa (accessed on 30 March 2026).

## Supplementary Materials

The following supporting information can be downloaded at: https://www.mdpi.com/article/10.3390/ani16081241/s1, Table S1: Behavioral effects of simulated typhoon stress on Wenchang chickens (mean ± SEM).

## Appendix A

**Table A1:** Real-time fluorescent quantitative PCR primer sequence list.

Gene	Forward Primer (5′-3′)	Reverse Primer (5′-3′)	Gene Sequence
*GHSR*	CCCGTATTCTGCCTCACGG	AAATACCACCACGACTAGCATC	NM_204394.2
*TAC1*	AGGCGTGAGGGACGTCAA	AGTAGCTGAGGTCATCGGGA	XM_004939318.5
*VIP*	CCAGAATTATTGATAGCTCCCAGG	TCGAAAGCGGCTGTAGTTGT	NM_001177309.2
*ATG5*	GGGTGCTTTCAGTTCCAGC	TGAAGCAGGTTGGTATGCGT	NM_001006409.2
*BECN1*	AGCAGGAAGAAGCTCAGTATCA	ACGCATCTGGTTCTCCACAC	NM_001006332.1
*CASP3*	AGCAAGTCTGTGGACTCTGG	GAACGAGATGACAGTCCGGT	XM_015276122.4
*TP53*	CGTTACCACGACGACGAGAC	GTACAGTCAGAGCCCACCTCG	NM_205264.1
*BCL2*	AGAGCGTCAACCGGGAGAT	TCCACAAAGGCATCCCATCCTC	NM_205339.3
*TJP1*	GCAGTCGTTCACGATCTCCT	TCTCTGCTTCGAAGACTGCC	XM_015278975.4
*CLDN5*	TGTCAGCCTTCATCGACGTG	TGGAATCGTACACCTTGCACT	NM_204201.2
*CLDN1*	GGTATGGCAACAGAGTGGCT	CAGCCAATGAAGAGGGCTGA	NM_001013611.2
*TNF-α*	TCAGACCAGATGGGAAGGGA	CAGTCTGAACTGTAACAGGTGTA	NM_204267.2
*IL6*	CAAGAAGTTCACCGTGTGCG	TCAGGCATTTCTCCTCGTCG	NM_204628.2
*OSTN*	TGACCTGCCTAAGAGACGGT	TTCAGAACAGAAGAACCCTTGGT	XM_025153364.2
*FSHR*	ATGTCTCCGGCAAAGCAAGA	CGTTCTCTGCAGGGCCAAA	NM_205079.2
*STAR*	CAGAGGGTTGGGAAGGACAC	GAACACTCACAAAGTCCCGC	NM_204686.3
*IGF1*	GAGTTGTGACCTGAGGAGGC	TTTGGCATATCAGTGTGGCG	NM_001004384.3
*MC4R*	CGGGAGGCTGCTATGAACAA	CATGGGCGAATGGAGGTTCT	NM_001031514.2
*ASS1*	GAATGACATCGCGAGCAAGC	GTACTTCCCGGTCCATGGTG	NM_001013395.2
*IL1RL1*	TAGAAGTGGAGCTTGGTGCTG	ATGGTCAAAGTTGCCTCTCCAT	NM_001024590.2
*FABP4*	GGAACCTGCTGGTGGAATGC	TGTGGAGTCTTTCTCCTTCAGTG	NM_204290.2
*TLR4*	TGGATCTTTCAAGGTGCCACA	AGTGTCCGATGGGTAGGTCA	NM_001030693.2
*CASP18*	AGCTTTCCAGTCCGTGTGTC	ACTCTCTTCAGAGCCTTTCCA	NM_001044689.2
*IRF7*	GGATCCGGCCAAATGGAAGA	TGTCATTGGGGACGCCTGAG	NM_205372.2
*GRP*	AGCTGGAAAGATGTGGTGGA	GGAAAGCGTTCCGGTTCTTC	NM_001277900.2
*WIF1*	TGTCCTTGCGCTCTTTGGAT	AACCCAACCTGAACCACTGA	NM_001199607.3
*GAPDH*	GGGTGGTGCTAAGCGTGTTA	GCACGATGCATTGCTGACAA	NM_204305.2
