# Supplementary material for: Effects of Simulated Typhoon Stress on Ovarian Function in Wenchang Chickens: An Exploration Based on the Microbiota–Gut–Brain–Ovarian Axis
Source: Animals (Basel). 2026 Apr 17;16(8):1241. doi: 10.3390/ani16081241 (PMC13113654; doi:10.3390/ani16081241)
Supplement: Supplementary file 1 [file animals-16-01241-s001.zip › animals-4185660-supplementary.pdf]

Table S1. Behavioral effects of simulated typhoon stress on Wenchang chickens (mean±SEM).

Items	Behavior Definition	Frequency												*P*-value
		Group T						Group C						
		2h	4h	6h	8h	10h	12h	2h	4h	6h	8h	10h	12h	
Feeding	Head oriented towards the trough with eating movements	1.54±0.36	0.33±0.33	0.33±0.21	0.17±0.17	0.00±0.00	0.00±0.00	1.20±0.28	0.50±0.22	0.00±0.00	0.33±0.21	1.00±0.36	0.83±0.48	0.3009
Drinking	Head inserted into the drinking trough with drinking movements	0.00±0.00	0.00±0.00	0.00±0.00	0.00±0.00	0.33±0.33	0.17±0.17	0.50±0.50	3.33±1.31	2.00±1.00	0.50±0.50	1.33±0.71	1.67±0.95	0.0022
Locomotion	Body spatial position changes	0.33±0.21	0.50±0.34	0.50±0.22	0.00±0.00	0.00±0.00	0.33±0.21	2.83±0.48	2.00±0.58	2.83±1.14	1.17±0.48	2.83±0.54	2.00±0.73	0.0004
Crouching	Tarsal joints or chest in contact with the cage floor	0.33±0.21	0.00±0.00	0.33±0.21	0.17±0.17	0.00±0.00	0.00±0.00	0.00±0.00	0.00±0.00	0.00±0.00	0.17±0.17	0.00±0.00	0.00±0.00	0.3030
Preening	Cleaning behavior of arranging feathers with the beak	0.00±0.00	1.17±0.48	1.50±0.56	1.33±0.49	1.33±0.49	1.67±0.49	0.00±0.00	0.67±0.33	1.17±0.83	1.50±0.50	1.00±0.45	1.33±0.67	0.3701
